# Semi-automated fabrication of customized ocular prosthesis with three–dimensional printing and sublimation transfer printing technology

**DOI:** 10.1038/s41598-019-38992-y

**Published:** 2019-02-27

**Authors:** JaeSang Ko, So Hyun Kim, Seung Woon Baek, Min Kyung Chae, Jin Sook Yoon

**Affiliations:** 1 Department of Ophthalmology, Severance Hospital, Institute of Vision Research, Yonsei University College of Medicine, Seoul, Republic of Korea; 2 Department of Biomedical Engineering, Yonsei University College of Medicine, Seoul, Republic of Korea

## Abstract

An ocular prosthesis is a custom-made polymeric insert that can be placed in an anophthalmic socket for cosmetic rehabilitation of patients who have lost their eyes. The process of creating such a custom-made ocular prosthesis is time-consuming and labor-intensive because it involves artistic work that is carried out manually. This paper proposes a novel semi-automated method for fabricating customized ocular prostheses using three-dimensional (3D) printing and sublimation transfer printing. In the proposed method, an impression mold of the patient’s anophthalmic socket is first optically scanned using a 3D scanner to produce a 3D model. The ocular prosthesis is then produced via a digital light processing 3D printer using biocompatible photopolymer resin. Subsequently, an image of the iris and blood vessels of the eye is prepared by modifying a photographed image of the contralateral normal eye, and printed onto the 3D-printed ocular prosthesis using a dye sublimation transfer technique. Cytotoxicity assessments of the base material and fabricated ocular prosthesis indicate that there is no adverse effect on cellular viability and proliferation. The proposed method reduces the time and skill required to fabricate a customized ocular prosthesis, and is expected to provide patients with easier access to quality custom-made ocular prostheses.

## Introduction

The removal of eyeballs is a well-established approach for the treatment of various ocular diseases, the leading indications being intraocular malignancy, severe trauma, and a painful blind eye^1^. Cosmetic rehabilitation using custom-made prosthetic devices in the form of ocular prostheses help individuals that undergo this procedure to gain professional and social acceptance and also alleviates other problems^2^. Ocular prostheses are custom-made because the location, size, contour, and color of each prosthesis have to be considered in order to provide realism and symmetry to anophthalmic patients^3^. In addition, custom-made, hand-painted, and individually constructed ocular prostheses have proven to be the most satisfactory ocular replacements. Customized fabrication, however, necessitates the service of skilled artists to duplicate the iris and sclera, and is a sophisticated and time-consuming process^4^. The process of creating such custom-made ocular prostheses involves manual artistic work, and there has been no significant improvement in the fabrication process over the past several decades^2–4^.

Three-dimensional (3D) printing is a process in which a product is built in a layer-by-layer fashion based on a computer-aided design (CAD) sketch^5^. This technology facilitates freeform design and the production of customized objects with complex geometries^5,6^. This type of printing is also being used in the medical field, where it is beginning to revolutionize medical and surgical procedures^7–10^. However, although 3D printing has recently been applied to the fabrication of ocular prostheses^11,12^, room for further development remains.

This paper proposes a semi-automated process for manufacturing customized ocular prostheses using 3D printing and surface painting techniques. The proposed process can provide patients with custom-made ocular prostheses without significant reliance on skilled ocularists.

## Methods

### 3D modeling of ocular prosthesis impression model

**Figure 1:**
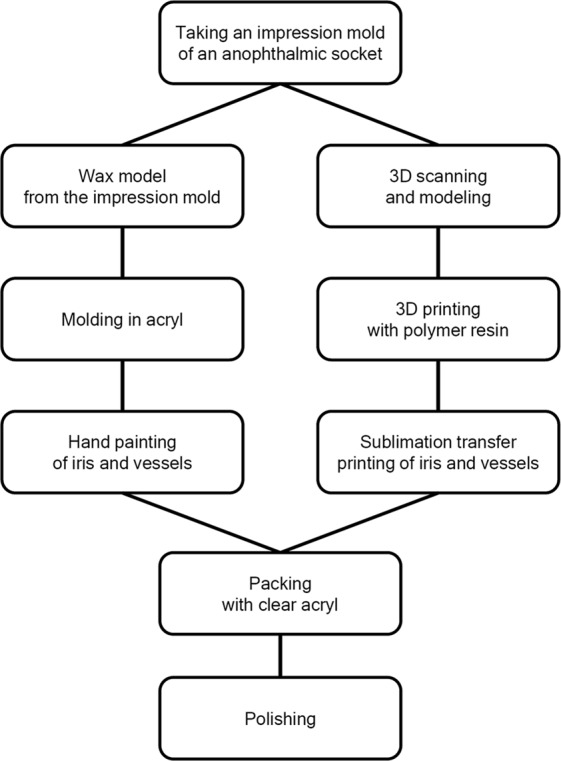
Protocol for manufacturing a custom-made ocular prosthesis: The conventional method and proposed semi-automated method with 3D printing technology are shown on the left and right, respectively.

The main steps in both the conventional method and the proposed semi-automated method for creating customized ocular prostheses are presented in Fig. 1. In the customized ocular prosthesis fabrication process, it is first necessary to obtain an impression of the target patient’s anophthalmic socket. Thus, a suitable amount of impression material was injected into the patient’s anophthalmic socket to determine the shape and size of the prosthesis. Comparing the contralateral normal fellow eye, the future iris center of the ocular prosthesis was marked on an impression mold. This was accomplished by one of the authors (S.W.B), who is an experienced ocularist.

Obtaining information about the size and shape of an object and creating 3D model data requires scanning technology. Various non-contact methods can be used to obtain 3D model data^13^, which can be produced based on computed tomography (CT) or magnetic resonance (MR) images^14^. However, because CT and MR images are intended for clinical use, special software is required to ensure that the data can be used for 3D modeling; in addition, their resolutions are limited. Alternatively, the light intensity (visual image) method, which illuminates a light source on a surface to obtain 3D model data directly, can be used^15^. In this study, we used a light intensity 3D scanner (Cara Scan 3.2, Kulzer Inc., Germany) that reflects the laser beam from the surface of an object to obtain 3D model data. The 3D scanner used a light source of 100 W, the camera resolution was 5 megapixels, the diagonal scanning range was 90–600 mm, and the precision was 5–12 µm. The impression model was scanned from top to bottom and bottom to top and the scans were merged using the program provided by the manufacturer. The iris center marking on the impression mold was optically scanned together with the mold and included in the 3D modeling data for future iris printing on 3D printed ocular prosthesis.

As the scanned image is produced from images scanned at multiple angles, the resultant image can include noise and artifacts, which inevitably had to be removed to produce the 3D model. This was achieved using the graphic editing software ZBrush 4R7 (Pixologic Inc., Los Angeles, CA, USA) to remove the internal and external noise of twisted meshes and to correct artifacts using the healing wizard function. Moreover, the software was used to smoothen the surface of the impression model. The polygon was spread evenly using the ZRemesher tool and the surface was smoothened using the polish brush tool to prevent the occurrence of a hole in the 3D model data. The modified 3D model data were converted to a stereolithography (STL) file and stored.

### 3D printing of the ocular prosthesis

The 3D printer used in this study was a DS131 (Carima Inc., Seoul, Republic of Korea), a digital light processing (DLP) 3D printer with 50-μm resolution in the X–Y plane. The Z-axis, that is, the layer thickness, can be set in 25-μm increments (i.e., 25 μm, 50 μm, and 75 μm). We used a biocompatible photopolymer resin (FotoTec DLP.A, Dreve Inc. Unna, Germany) as the base material. Of the various colors available for the material, white was selected because it most closely approximates the color of the sclera.

As the viscosity of the biocompatible material used was higher than the viscosity recommended by the manufacturer of the 3D printer, the reduced adhesion proved problematic. The printing stability was improved by attaching a heating plate to the tray containing the liquid resin in the 3D printer, thereby reducing the viscosity of the material. Subsequently, the curing tendency of the resin was analyzed, and its thickness was measured to specify an appropriate amount of light for the molding time based on the characteristics of the material.

After the 3D printer was set up, a slicing file was created with the complete 3D model data using the software provided by the manufacturer. This file was uploaded to the 3D printer and the ocular prosthesis was printed. The ocular prosthesis was then cleaned with alcohol to remove residual resin remaining on its surfaces and the supports were removed. The prosthesis was then cured at 170 °C for 30 min and post-cured at 100 °C for 2 h.

### Printing iris and blood vessels on the printed ocular prosthesis

The contralateral normal eye of the patient was photographed using a slit lamp biomicroscope (Haag-Streit AG, Koeniz, Switzerland) with a mounted digital camera (Canon 450D, Canon Inc. Tokyo, Japan) to capture the graphical data of the iris and blood vessels. The image was copied using a mirroring tool and the blood vessels were modified by maintaining the size of the iris at 11 mm. Then, parts of the image containing the pupil, iris, and blood vessels were selected and the brightness and contrast of the image were modified using Photoshop CS4 (Adobe Systems Inc., San Jose, CA, USA) to produce an image file for printing.

**Figure 2:**
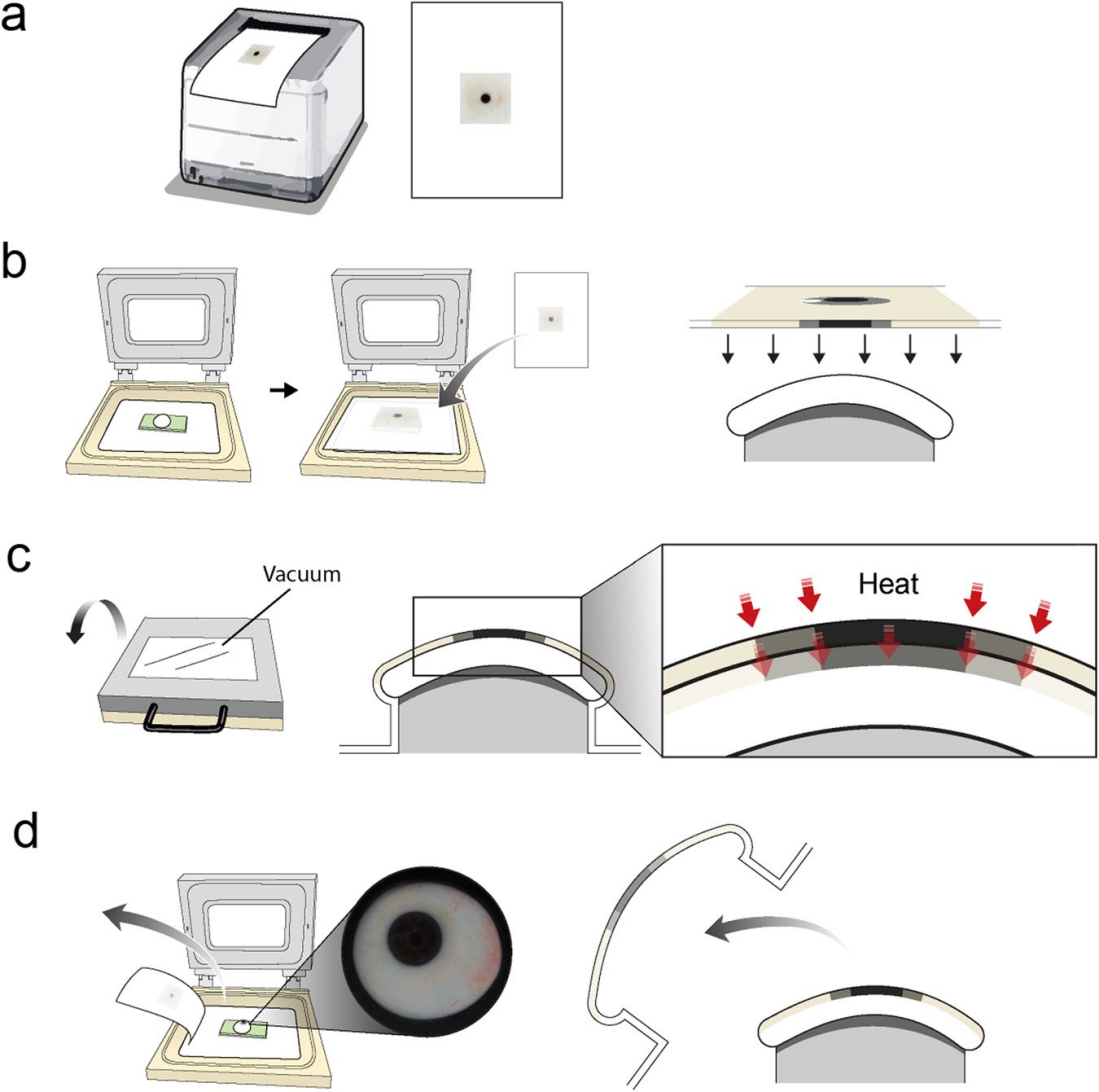
Schematic representation of the steps comprising the sublimation transfer technique (**a**) The image of the iris and blood vessels is printed on transfer paper via an inkjet printer using biocompatible sublimation dye. (**b**) A frame made to fit the back curvature of the ocular prosthesis is placed on the sublimation transfer system, and the 3D-printed ocular prosthesis placed on the frame. Then the printed transfer paper is placed upside down. (**c**) Vacuum is applied so that the transfer paper and the 3D-printed output are in contact, then heat is applied. (**d**) After the printed sublimation ink is transferred onto the 3D-printed output and fixed, the transfer paper is removed. The authors would like to thank Dong-Su Jang, MFA (Medical Illustrator, Medical Research Support Section, Yonsei University College of Medicine, Seoul, Korea) for his help with this illustration.

The sublimation transfer technique was used to print the image of the iris and blood vessels on the 3D-printed ocular prosthesis. An inkjet printer (Epson Stylus PRO 7890, Seiko Epson, Nagano, Japan) was used to print the image on the transfer paper (Seiko Epson, Nagano, Japan) with biocompatible sublimation ink (SubliNova Smart, Inktec Inc., Witney, UK). The iris and blood vessels printed on the transfer paper were transferred to the 3D-printed ocular prosthesis using a sublimation transfer system optimized for curved surface printing (3D STAR–6 S, Diofun Inc., Gunpo, Republic of Korea) after the system was preheated to 130 °C in accordance with the manufacturer’s protocol. The steps in the sublimation transfer technique are schematically illustrated in Fig. 2.

### Evaluation of the cellular toxicity of the 3D-printed ocular prosthesis

The cytotoxicity of the materials used for 3D printing was evaluated using the method described in our previous study^16^. Specifically, samples of 3D-printed polymer resin were prepared in the form of 1 × 1 × 1 cm blocks, and samples of poly(methyl methacrylate) (PMMA)—the material used to fabricate most modern ocular prostheses—were prepared in the same shape and size. Then, the cytotoxicity was assessed by collecting the solutions that leached from the samples following the recommendations of the International Organization for Standardization (ISO) 10993-12 using cell culture medium. The cell viability was assessed using a 3-(4,5-dimethylthiazol-2-yl)-5-(3-carboxymethoxyphenyl)-2-(4-sulfophenyl)-2H-tetrazolium (MTS) assay in L929 cells in accordance with the manufacturer’s protocol (Promega Corp., Madison, WI, USA).

After evaluating the cytotoxicity of the material, the cytotoxicity of the final product was assessed in accordance with the ISO 10993-5 recommendations. The ocular prosthesis fabricated using the conventional protocol was also tested for negative control. The solution leached from the ocular prosthesis was prepared using a ratio of 4 g/20 mL with the cell culture medium at 37 °C for 24 h. This involved culturing 1 × 10^5^ cells/mL of the L929 cells in the leached solution for 1, 3, and 5 days before they were assessed with live/dead cell assay (Thermo Fisher Scientific, Rockford, IL, USA) in accordance with the manufacturer’s protocol. The results were quantified using fluorescence-activated cell sorting analysis.

## Results

### 3D modeling of the ocular prosthesis impression model

**Figure 3:**
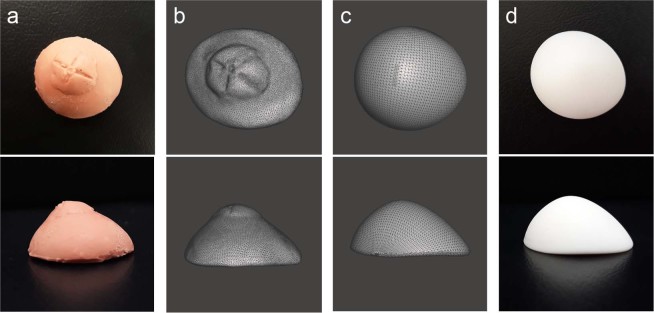
Three-dimensional (3D) model of impression mold of patient’s anophthalmic socket and 3D printed output. (**a**) Impression mold of patient’s anophthalmic socket obtained by following the conventional protocol for manufacturing an ocular prosthesis. (**b**) Optical scan of the impression mold using a 3D scanner, and (**c**) modified and smoothened image to reduce artifacts. (**d**) 3D printed output of ocular prosthesis printed using a DLP printer.

Obtaining a 3D scan of the impression mold required approximately 30 min. Further, another 30 min period was necessary to modify the 3D scan data using graphic software. By contrast, crafting a wax model from the impression mold requires a similar amount of time—approximately 1 h—in the conventional method. Although the same amount of time is necessary to complete both procedures, 3D scanning is an automated process that does not require human effort. Moreover, a database of patients’ ocular prostheses can be built using the software. An impression mold of a patient’s anophthalmic socket, 3D scanned image data, and its modified image are shown in Fig. 3(a–c).

### 3D printing of the ocular prosthesis

A DLP printer needs to be set up using custom settings for the luminance and amount of light to match the characteristics of the target material in order to obtain an optimal output. The 3D printer utilized was originally designed to use a base material with viscosity of 100–120 mPa·s. Thus, as the biocompatible material used in this study had a higher viscosity of 680–720 mPa·s, the adhesion of the printing material was low, thereby causing the printout to disintegrate during printing. Consequently, printing was stabilized by attaching a heating plate to the tray to maintain a temperature range of 50–60 °C. The technical parameters of the 3D printer are provided in Table 1 and the printed ocular prosthesis is shown in Fig. 3(d). Printing the 3D model of the ocular prosthesis took approximately 45 min, as opposed to manually creating an acrylic mold following the conventional process, which required approximately 1 h. The 3D printed ocular prosthesis weighed approximately 2 g.

**Table 1:** Technical parameters of the 3D printer used in this study.

Layer thickness	50 μm
XY resolution	50 μm
Energy density	3.0 mW/cm^2^
Initial exposure time (three layers)	30 s
Basic exposure time	2 s

### Printing the iris and blood vessels on the printed ocular prosthesis

**Figure 4:**
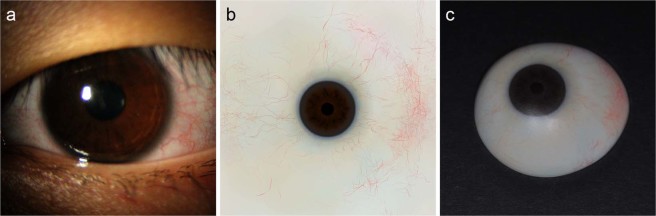
Printing iris and blood vessels onto printed ocular prosthesis (**a**) The contralateral normal eye was photographed using a slit lamp. (**b**) Image of contralateral normal eye modified for printing. (**c**) Image of iris and blood vessels transferred to 3D printed ocular prosthesis.

**Figure 5:**
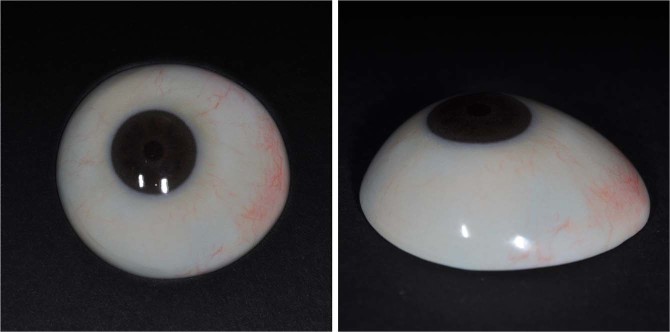
Final output of the customized ocular prosthesis fabricated using 3D printing and surface printing.

The contralateral normal eye of the patient was photographed using a slit lamp to create graphical data of the iris and blood vessels (Fig. 4(a)). Parts of the image containing the pupil, iris, and conjunctival vessels were selected and the brightness and contrast of the image were modified to produce an image file for printing (Fig. 4(b)). The modified image of the iris and blood vessels was then printed on transfer paper, and transferred to the 3D-printed output of the ocular prosthesis using a dye-sublimation transfer system (Fig. 4(c)). The process of capturing the image of the contralateral normal eye and graphical modifications required approximately 30 min, and printing the image on transfer paper and sublimating it onto the ocular prosthesis required another 30 min. By contrast, manually painting the iris and blood vessels usually requires more than 3 h, even when the process is performed by a skilled ocularist. Finally, the ocularist applied a transparent PMMA coating to the 3D printed ocular prosthesis to complete the fabrication process (Fig. 5).

### Cytotoxicity assessment

**Figure 6:**
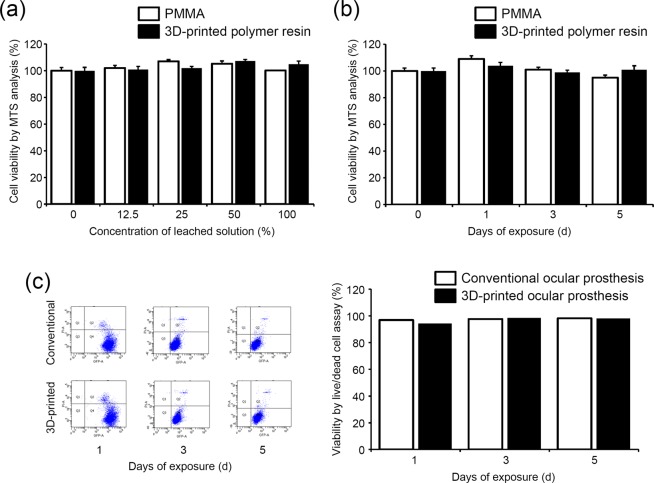
Effect of fabricated ocular prosthesis on cell viability. MTS assay using the solution leached from the PMMA and 3D-printed polymer resin used for 3D printing. (**a**) L929 cells were cultured in different concentrations of leached solution for 48 h, and the cell viability was measured with MTS analysis. (**b**) L929 cells were cultured in 100% of leached solution for different durations, and the cell viability was measured with MTS analysis. (**c**) The cytotoxicity of the final products was assessed with live/dead cell assay and quantified using fluorescence-activated cell sorting analysis.

The cytotoxicity of the solution leached from each sample was evaluated using cell viability test because the ocular prostheses are in constant contact with the conjunctiva of the wearers’ anophthalmic sockets. As shown in Fig. 6(a,b), the solutions leached from the 3D-printed polymer resin did not decrease the viability of the L929 cells in the MTS analysis.

Moreover, neither the ocular prosthesis fabricated using the conventional method nor that created via our semi-automated process resulted in significant cytotoxicity in live/dead cell assay (Fig. 6(c)). After 1, 3, and 5 days of incubation in the leached solution from both ocular prostheses, the ratios of live/dead cell were not significantly decreased.

## Discussion

Conventional custom-made ocular prostheses need to be designed and manually fabricated by skilled ocularists. As this involves several manual processes, it is time-consuming, labor-intensive, and expensive^2,12,17^. In addition, as it is not possible to store data pertaining to a previously created ocular prosthesis, reproducing the original product in the case of damage or loss would require the same amount of time, effort, and cost. The semi-automated method proposed in this study replaces the manual processes in the conventional method (specifically, building a wax model for the socket, molding the ocular prosthesis in acryl, and painting the iris and blood vessels by hand) with semi-automated processes (specifically, 3D scanning and modeling, 3D printing of the ocular prosthesis, and sublimation transfer printing), as shown in Fig. 1. In contrast to the conventional custom ocular prosthesis fabrication process, which requires approximately 10 h^12^, custom ocular prosthesis fabrication using the proposed process requires approximately 8 h. Although the amount of time saved in the fabrication process is not large, much of the process is replaced by semi-automated processes that do not require manual labor by a skilled craftsman. Thus, the proposed process only requires approximately 3 h of actual work for a manufacturer. Moreover, one ocularist can fabricate ocular prostheses of multiple patients at the same time, thereby increasing the efficiency of ocular prosthesis fabrication and ultimately reducing the cost of fabricating an ocular prosthesis. In addition, a relatively inexperienced ocularist or a non-professionally educated general worker can use the semi-automated process to create a custom-made ocular prosthesis, which can improve the welfare of anophthalmic patients in underdeveloped areas where experienced ocularists are not available. Furthermore, because the ocularist can store information about patients’ ocular prostheses, the ocular prosthesis can be reproduced without additional visit to the practice in cases where patients have lost or damaged their ocular prosthesis.

Various types of 3D printing technology have been developed for industrial use, some of which have been adopted to produce medical devices^5,8,18^. Stereo lithography (SLA)-type 3D printing uses a UV light source to selectively liquidize a photopolymer, whereas fused deposition modeling (FDM) extrudes semi-liquid plastic in a specific layout to create objects. The most significant advantage of SLA 3D printing is its high resolution: SLA 3D printing can produce objects with more than twice the resolution of FDM printing^19,20^. Similarly, polyjet technology relies on the principle of UV curing, but the liquid photopolymer is deposited onto a build platform in the form of droplets, and each droplet corresponds to a voxel that constitutes the 3D object^21,22^. In DLP-type 3D printing, the digital optical projector immediately irradiates a cross-sectional layer using a photocurable resin and repeatedly stacks layers as its output. Unlike the SLA approach of photo-curing based on points, DLP performs photo-curing based on units of cross-sectional layers; this results in faster fabrication and hardened products with a smooth surface^23–25^.

The challenge in manufacturing a medical device with a 3D printer lies in selecting an appropriate material because the material must be biocompatible and the printed output is usually required to have a smooth and homogenous surface. Some biomaterials available for 3D printing usually produce an inconsistent output owing to their high viscosities below a certain temperature. We solved this problem by installing a heating plate in the 3D printer to raise the temperature during printing, which lowered the viscosities and improved the quality of printing.

Several methods have been developed for printing on curved surfaces, such as direct inkjet printing, laser printing, pigment transfer printing, and dye-sublimation transfer printing^26–28^. Because of the marked curvature of the ocular prosthesis, the dye-sublimation transfer method was used in this study^29–31^. As this method transfers the printed images from the transfer paper to the curved surface of the product by penetrating the colors at a certain temperature and pressure, the design is neither damaged nor peeled^32,33^. Thus, this method is used for printing on cotton fabrics and can be used for products with curved surfaces, such as cell phone cases, glasses, and glass cups.

Several recent studies that utilize 3D printing technology to manufacture custom-made ocular prostheses have been reported. For example, Ruiters *et al*. produced a 3D printed mold, instead of impression molds, by CT imaging in anophthalmic patients^11^. Their technique avoids the process of fabricating the impression mold, which patients may find uncomfortable. However, it has several drawbacks. First, it entails replacing the impression mold with a 3D-printed device, and the ocular prosthesis itself is manufactured by hand, as in the conventional method. Second, fabricating the impression mold using CT imaging can be inaccurate. This is because the anophthalmic cavity is collapsed and the boundaries of the anophthalmic socket are not well distinguished with CT scans when there is no prosthesis. Moreover, as their technique cannot capture the anterior curve of the anophthalmic socket, it is inappropriate to use the term “custom-made” because the mean standard values of a normal eye are used instead.

Alam *et al*. recently proposed a method of fabricating ocular prostheses with polyjet 3D printing using PMMA^12^. They reported that the proposed method reduces the fabrication time while maintaining a satisfactory cosmetic outcome. However, as with the conventional method of ocular prosthesis fabrication, it produces a wax model from an impression and records a CT scan of the model to obtain a 3D model for 3D printing. Considering that an acrylic mold can be manufactured directly from the wax model in the conventional ocular prosthesis fabrication method, substituting it with CT scanning followed by 3D printing does not appear to be highly advantageous. Moreover, the authors also manually painted the iris and blood vessels, which requires a skilled professional.

The primary limitation of the method proposed in this paper is that certain aspects of the manufacturing process still need to be undertaken by a skilled ocularist. Specifically, expert manual work is required to obtain an impression mold of the patient’s anophthalmic socket and to cover the printed ocular prosthesis with clear acrylic coating and polish the coating. These manual tasks are still necessary to create a customized ocular prosthesis for patients. Nevertheless, as much of the production process is replaced by a semi-automated process, the time required for production is significantly reduced and the need for technical expertise is reduced. In addition, this technique requires skills as well as software for 3D graphics and modeling, which were not previously required.

Because the present study was only concerned with the development of a prototype for the manufacturing process, it did not include a human trial. Therefore, the cosmetic outcome and the patient’s comfort when wearing the fabricated ocular prosthesis could not be measured. We plan to conduct clinical trials for the proposed method and publish the results in the near future.

In summary, this paper proposed a novel semi-automated method for fabricating customized ocular prostheses that reduces the time and skill required to fabricate customized ocular prostheses. The fact that the 3D modeling data can be stored is also advantageous as the data can be reused when the patient’s ocular prosthesis is lost or damaged. The proposed method makes it easier for patients to access quality custom-made ocular prostheses, which helps them to gain increased professional and social acceptance.
